# Cost effectiveness of a vascular access education and training program for hospitalized emergency department patients

**DOI:** 10.1371/journal.pone.0310676

**Published:** 2024-10-01

**Authors:** Amit Bahl, Yuying Xing, S. Matthew Gibson, Emily DiLoreto

**Affiliations:** 1 Department of Emergency Medicine, Corewell Health William Beaumont University Hospital, Royal Oak, Michigan, United States of America; 2 Oakland University William Beaumont School of Medicine, Rochester, Michigan, United States of America; 3 Corewell Health Research Institute, Royal Oak, Michigan, United States of America; 4 Vascular Access Consulting, Henderson, Kentucky, United States of America; Jordan University of Science and Technology Faculty of Nursing, JORDAN

## Abstract

**Objective:**

Education and training in vascular access is a critical component to delivering quality vascular access care. Given that organizations must invest resources to implement and sustain high-quality vascular access programming, we aimed to demonstrate the cost effectiveness of a program (Operation STICK (OSTICK)) in the emergency department (ED).

**Methods:**

This was an observational cohort study conducted at a tertiary care academic center with 120,000 ED visits. Consecutive hospitalized adults with ultrasound-guided (DIVA) and traditionally-placed (non-DIVA) peripheral intravenous catheters (PIVC) in the ED were included in the analysis. Two groups (OSTICK and non-OSTICK) were compared in the analysis: OSTICK PIVCs were inserted by clinicians with formal, standardized training in peripheral venous access while non-OSTICK PIVCs were inserted by staff with basic departmental training in PIVC care. Cost factors included number of procedures, wait time to establish a PIVC, complications, and training. Effect was complication-free PIVC functionality. Multiple linear regressions were used to estimate incremental cost (ΔC), incremental effect (ΔE), and incremental net benefit (INB) of the OSTICK program.

**Results:**

From 10/1/2022 thru 3/31/2023, 21,259 PIVCs including 1681 OSTICK and 19,578 non-OSTICK PIVCs were included in the analysis. Average age was 64.8 and 53.7% were female. The estimate of incremental cost (ΔC) for each patient was -$83.175 (95% CI: -$103.953 to -$62.398; p<0.001), indicating that the OSTICK group saves money compared to the non-OSTICK group. The OSTICK group is also more effective at increasing the proportion of catheter dwell time relative to hospital length of stay (ΔE), with an estimate of 0.037 (95% CI: 0.016 to 0.059; p<0.001), compared to those in the non-OSTICK group. The estimated incremental cost-effectiveness ratio (ICER) for the OSTICK group compared with the non-OSTICK group was −$221.964 (95% CI: -$177.400 to -$381.716) per ten percentage points of PIVC dwell time to hospital length of stay increase.

**Conclusions:**

Strategic investment in vascular access education and training can yield impressive financial returns while simultaneously enhancing vascular access outcomes. It is imperative for organizations to recognize the significant impact of such initiatives and prioritize the implementation of comprehensive programs.

## Introduction

Peripheral intravenous catheter (PIVC) placement is one of the most routine invasive procedures performed on hospitalized patients daily with 2 billion PIVCs used every year across the globe [1, 2]. Despite its pervasive use, peripheral vascular access care is fraught with complications and failure in the majority of insertions [3]. As patients require functional vascular access for treatment, they are often subject to multiple insertions over the course of hospitalization to counter these inadequacies [4]. Further, as up to one-third of patients have difficult vascular access (DIVA) [5], obtaining and maintaining access in these patients can be particularly challenging requiring specialty teams and advanced vascular access devices [6]. The escalation process is typically cumbersome and is often linked to more complications, treatment delays, and extended hospital stays [7].

Beyond poor patient outcomes, PIVC inadequacies contribute to an enormous, unnecessary financial burden to the tune of $1.5 billion USD annually [7, 8]. Curbing healthcare costs related to vascular access care is a critical strategy to reduce costs as functional vascular access is required for many critical therapies such as fluid infusions, medications, and blood products across all departments within the hospital and used for a broad range of inpatient diagnoses [9]. Despite development and dissemination of numerous technologies to improve vascular access outcomes, there has been limited improvement in outcomes and therefore the heavy cost burden remains [3, 10]. Notably, one area of focus that has shown broad improvement in PIVC outcomes is educational interventions [11]. Specifically, emergency department (ED) based interventions that target DIVA patients have shown to be very effective in improving complication-free dwell [12, 13]. As the ED is the gateway to nearly 70% of all hospital admissions, the potential downstream impact of better vascular access care is compelling [12, 14, 15]. While the impact on patient outcomes is proven, educational interventions require long and short-term resources and can be costly undertakings. Therefore, the aim of this study is to investigate if broad ED-based education and training in vascular access is a cost-effective solution for hospitalized patients.

## Materials and methods

### Study design, setting participants

This was a retrospective, observational, cohort study conducted at a tertiary care academic center in metropolitan Detroit. The facility has 1100 hospital beds and 160 ED beds. Nearly half of all inpatient admissions arise from the ED. Consecutive adult patients requiring inpatient admission with documentation of a PIVC placed in the ED were eligible participants. Data were accessed 6/15/2023. Corewell Health Institutional Review Board (IRB) approved this investigation. Due to retrospective nature of the study design and large volume of subjects, the IRB waived the informed consent requirement.

### Resource use, variables, measurements

All data was gathered from the electronic health record (EHR) (EPIC, Verona, Wisconsin). Data variables included patient factors and PIVC characteristics. Patient factors included demographics, BMI, Charlson Comorbidity Index (CCI), and Emergency Severity Index (ESI). PIVC characteristics included: insertion technique: ultrasound-guided = DIVA; traditional = non-DIVA, gauge, orientation, location, attempts, PIVC insertion and removal instances, and complications.

The cost model included the following parameters: IV wait time, number of procedures, complications, and training. IV wait time was defined as time from registration in the ED until IV was established and documented in the EHR. Number of procedures was defined as the number of attempts to successfully place the index PIVC. Complications included occlusion, dislodgment, infiltration, and phlebitis and were based on daily clinician assessment of the PIVC site. Training costs included costs associated with the delivery of the education and training for the Operation STICK intervention.

The cost associated with IV wait time was calculated using two approaches: 1. Centers of Disease Control and American Hospital Association’s published costs regarding average cost per hospitalization [16–18] and 2. Expert opinion of the Chief Financial Officer at a major health system [19]. As number of attempts was not documented prior to the OSTICK program and only documented by a limited number of non-OSTICK trained staff thereafter, this data point was not available for the non-OSTICK group, Instead, the number of attempts for non-OSTICK was based upon the best available literature [20]. The cost of PIVC insertion included cost of materials and labor and was estimated based on existing literature [21, 22]. The costs associated with complications included removal and replacement of the PIVC and treatment of the complication as needed. These costs were based on existing literature [7]. Finally, the costs of training include the cost of the trainee (meshed cost of ED nurse and ED technician) as well as estimated salary of a nurse educator. Table 1 illustrates the costs used in this model.

**Table 1:** Itemized list of factors and costs related to PIVC education, insertion, and maintenance.

Factors	Costs
**PIVC Wait Time**	
• AHRQ Publication	$120.13/hour
• Expert Opinion	$20.83/hour
**PIVC Procedure**	
• Material + Labor	$35/traditional & $45/US-guided
**Complications**	
• Infiltration	$45 for replacement PIVC in 70%
	$40 for hot compress in 80%
• Occlusion	$45 for replacement of new catheter in 80%
**OSTICK Training**	
• Educator	$8333/month
• Trainee	$3360/month

### Operation STICK vascular access program overview [12–14]

OSTICK is a formalized, evidenced-based, comprehensive vascular access training program that standardizes the assessment, preparation, insertion, and documentation of peripheral vascular access for basic and advanced placements. The program balances the clinical need for better outcomes with a shrinking pool of resources by elevating nurses and ED technicians to provide specialty-level vascular access care. It incorporates the use of standardized assessment tools, education, and training of PIVC placement as well as utilizing cutting-edge products, advanced technologies, optimized inventory control, quality infection prevention practices, and real-time data tracking for surveillance. This program began in October 2021 and has trained 160 clinicians, with 136 achieving competency through March 2023. 79 (49.4%) are nurses, 62 (38.8%) are ED technicians, and 19 (11.9%) have other credentials (e.g. physician, advanced practice provider, or medical student).

Education and training in OSTICK follows a consistent and sequential path: all trainees participate in a pre-workshop video didactic series, in-person workshop with simulation, and precepted bedside training. The pre-workshop modules provide an overview of venous access, including a review of assessment strategies, infection prevention, basic and advanced insertion techniques, equipment, supplies, documentation, and example cases with real-time feedback. The in-person workshop consists of a didactic lecture and PIVC insertion exercises and simulation. The didactic coursework reinforces key concepts and assessment strategies from the didactic video series through the integration of simulation training. Finally, the one-on-one precepting sessions individualized practice on vein blocks, followed by supervised PIVC placement on ED patients with minimal instruction by the preceptor. These insertions are assessed using a comprehensive checklist of the steps for PIVC insertion using OSTICK principles. Once a trainee can complete all steps sequentially without prompting, they are considered competent. The expectation is for the clinician to safely insert a PIVC with an 80% placement success rate in 1–2 insertion attempts.

Starting in 2014, several ED staff (primarily physicians, advanced practice providers, and some nurses) were trained in US PIVC placement at the study institution with a much narrower focus. This training included a didactic workshop (3 h) and a requirement to log 10 successful placements to attain competency. Once OSTICK was developed in October 2021, this training was discontinued.

### Statistical analysis

Descriptive analysis was conducted to provide an overview of the clinical characteristics. Continuous variables were summarized using means and standard deviations. Categorical variables were presented as frequencies and percentages. The T-test was used to compare continuous variables between two groups, while the Chi-square test was employed for categorical variables.

For the Cost-effectiveness analysis (CEA) comparing the OSTICK group versus the non-OSTICK group, regression-based methods were employed [23, 24]. Multiple linear regressions were used to estimate incremental cost (ΔC), incremental effect (ΔE), and incremental net benefit (INB) of the OSTICK program (S1 Table). Age, race, gender, BMI, CCI, ESI, and insertion method were included as potential confounders due to the statistically significant differences between the two groups. The incremental cost-effectiveness ratio (ICER) was calculated as $\frac{\Delta C}{\Delta E}\times 0.1,\;$ representing the extra cost per ten percentage points of PIVC dwell time to hospital length of stay. The individual’s net benefit was calculated as *WTP* × *E*_*i*_ − *C*_*i*_. The cost-effectiveness acceptability curve (CEAC) was employed to characterize the relationship between cost-effectiveness and the willingness to pay (WTP), formed using p-values from INB regressions [25].

The estimated results were reported with corresponding 95% CIs and p-values for the multiple linear regression analysis. All statistical tests were two-sided, and statistical significance was determined using a p-value threshold of less than 0.05. The analysis was conducted using R-4.3.1, provided by the R Foundation for Statistical Computing.

## Results

From 10/1/2022 thru 3/31/2023, 21,2159 patients were included of which 1660 were DIVA and 19599 were non-DIVA. Within DIVA, 989 were OSTICK and 671 non-OSTICK. Average age was 62.6, 64.1% female, and 52.5% were Black. Number of attempts were higher for non-OSTICK (1.7) compared to OSTICK (1.27); p<0.001. IV wait time was longer for non-OSTICK (4.17 hours) than OSTICK (3.02 hours); p<0.001. (Table 2) Within non-DIVA, 692 were OSTICK and 18907 were non-OSTICK. Average age was 65, 52.8% female, and 66.2% were White. Number of attempts were higher for non-OSTICK (1.37) compared to OSTICK (1.15); p<0.001. IV wait time was similar for non-OSTICK (1.99 hours) and OSTICK (1.80 hours); p = 0.071 (Table 3).

**Table 2:** Patient characteristics, PIVC characteristics, PIVC outcomes, and PIVC cost for DIVA patients.

Variables*	All	Non-OSTICK	OSTICK	*p* value
n	1660	671 (40.4%)	989 (59.6%)	
**Patient Characteristics**				
Age, years				<0.001^1^
Mean	62.61 (18.05)	60.45 (18.29)	64.08 (17.75)	
Age Group				0.012^2^
18–64	816 (49.2%)	358 (53.4%)	458 (46.3%)	
65–80	563 (33.9%)	215 (32.0%)	348 (35.2%)	
80+	281 (16.9%)	98 (14.6%)	183 (18.5%)	
Sex				0.538^2^
Female	1064 (64.1%)	436 (65.0%)	628 (63.5%)	
Male	596 (35.9%)	235 (35.0%)	361 (36.5%)	
Race				0.002^2^
Black or African American	871 (52.5%)	382 (56.9%)	489 (49.4%)	
White or Caucasian	718 (43.3%)	256 (38.2%)	462 (46.7%)	
Other	71 (4.3%)	33 (4.9%)	38 (3.8%)	
BMI, kg/m^2^ (n = 1659/670/989)				0.779^1^
Mean	30.43 (9.36)	30.35 (9.32)	30.48 (9.39)	
Charlson Comorbidity Index				0.353^2^
0	541 (32.6%)	206 (30.7%)	335 (33.9%)	
1–2	523 (31.5%)	206 (30.7%)	317 (32.1%)	
3–4	308 (18.6%)	129 (19.2%)	179 (18.1%)	
> = 5	187 (11.3%)	84 (12.5%)	103 (10.4%)	
N/A	101 (6.1%)	46 (6.9%)	55 (5.6%)	
Emergency Severity Index (n = 1656/668/988)				0.303^1^
Mean	2.52 (0.53)	2.51 (0.54)	2.53 (0.53)	
**PIVC Characteristics**				
Gauge				0.036^2^
18	45 (2.8%)	26 (4.0%)	19 (1.9%)	
20	1549 (95.0%)	607 (93.5%)	942 (96.0%)	
22	36 (2.2%)	16 (2.5%)	20 (2.0%)	
Not documented	30	22	8	
Orientation		45 (2.8%)		0.558^2^
Left	882 (53.4%)	349 (52.5%)	533 (53.9%)	
Right	771 (46.6%)	316 (47.5%)	455 (46.1%)	
Not documented	7	6	1	
Location				<0.001^2^
Upper arm	822 (49.5%)	303 (45.2%)	519 (52.5%)	
Antecubital	196 (11.8%)	135 (20.1%)	61 (6.2%)	
Forearm	631 (38.0%)	224 (33.4%)	407 (41.2%)	
Hand/Wrist	2 (0.1%)	2 (0.3%)	0 (0.0%)	
Other	9 (0.5%)	7 (1.0%)	2 (0.2%)	
Not documented	0	0	0	
**PIVC Outcomes**				
Prop Dwell to Stay				0.006^1^
Mean	0.65 (0.37)	0.62 (0.37)	0.67 (0.37)	
IV attempts				<0.001^1^
Mean	1.44 (0.49)	1.70 (0.00)	1.27 (0.57)	
IV wait time				<0.001^1^
Mean	3.49 (4.66)	4.17 (6.62)	3.02 (2.50)	
Complications				0.744^2^
Infiltration/Leaking	273 (72.2%)	126 (71.6)	147 (72.8)	
Occlusion	105 (27.8%)	50 (28.4%)	55 (27.2%)	
Not documented	1282	495	787	
**PIVC Cost ($)**				
IV attempts				<0.001^1^
Mean	64.90 (21.97)	76.50 (0.00)	57.02 (25.64)	
IV wait time low				<0.001^1^
Mean	72.58 (97.07)	86.77 (137.80)	62.96 (52.11)	
IV wait time high				<0.001^1^
Mean	418.61 (559.82)	500.44 (794.72)	363.09 (300.51)	
Complications				0.009^1^
Mean	12.72 (24.16)	14.61 (25.33)	11.44 (23.26)	
OSTICK training cost				<0.001^1^
Mean	0.70 (0.57)	0.00 (0.00)	1.17 (0.00)	
Total cost low				<0.001^1^
Mean	150.90 (103.51)	177.88 (138.93)	132.59 (63.81)	
Total cost high				<0.001^1^
Mean	496.92 (561.93)	591.54 (793.93)	432.72 (304.25)	

Abbreviations: BMI = body mass index; PIVC = peripheral intravenous catheter

*For continuous variables, mean (standard deviation) were presented. For categorical variables, frequencies (percentage) were presented.

^1^T-test

^2^Chi-square test

**Table 3:** Patient characteristics, PIVC characteristics, PIVC outcomes, and PIVC cost for Non-DIVA patients.

Variables*	All	Non-OSTICK	OSTICK	*p* value
n	19599	18907 (96.5%)	692 (3.5%)	
**Patient Characteristics**				
Age, years				<0.001^1^
Mean	64.93 (18.63)	64.93 (18.65)	64.92 (18.30)	
Age Group				0.840^2^
18–64	8425 (43.0%)	8133 (43.0%)	292 (42.2%)	
65–80	6952 (35.5%)	6707 (35.5%)	245 (35.4%)	
80+	4222 (21.5%)	4067 (21.5%)	155 (22.4%)	
Sex				0.466^2^
Female	10349 (52.8%)	9993 (52.9%)	356 (51.4%)	
Male	9250 (47.2%)	8914 (47.1%)	336 (48.6%)	
Race				0.053^2^
Black or African American	5208 (26.6%)	4999 (26.4%)	209 (30.2%)	
White or Caucasian	12980 (66.2%)	12551 (66.4%)	429 (62.0%)	
Other	1411 (7.2%)	1357 (7.2%)	54 (7.8%)	
BMI, kg/m^2^ (n = 19576/19884/692)				0.480^1^
Mean	28.62 (8.05)	28.63 (8.04)	28.41 (8.20)	
Charlson Comorbidity Index				0.014^2^
0	8544 (43.6%)	8219 (43.5%)	325 (47.0%)	
1–2	4792 (24.5%)	4650 (24.6%)	142 (20.5%)	
3–4	2261 (11.5%)	2190 (11.6%)	71 (10.3%)	
> = 5	1281 (6.5%)	1221 (6.5%)	60 (8.7%)	
N/A	2721 (13.9%)	2627 (13.9%)	94 (13.6%)	
Emergency Severity Index (n = 19543/18854/689)				<0.001^1^
Mean	2.48 (0.54)	2.48 (0.54)	2.55 (0.54)	
**PIVC Characteristics**				
Gauge				0.002^2^
18	2141 (11.6%)	2083 (11.7%)	58 (8.5%)	
20	15348 (82.9%)	14743 (82.7%)	605 (88.3%)	
22	1019 (5.5%)	997 (5.6%)	22 (3.2%)	
24	4 (0.0%)	4 (0.0%)	0 (0.0%)	
Not documented	1087	1080	7	
Orientation				0.270^2^
Left	9547 (49.2%)	9193 (49.1%)	354 (51.2%)	
Right	9870 (50.8%)	9533 (50.9%)	337 (48.8%)	
Not documented	182	181	1	
Location				<0.001^2^
Upper arm	911 (4.6%)	847 (4.5%)	64 (9.2%)	
Antecubital	10806 (55.1%)	10522 (55.7%)	284 (41.0%)	
External Jugular	14 (0.1%)	14 (0.1%)	0 (0.0%)	
Foot	6 (0.0%)	6 (0.0%)	0 (0.0%)	
Forearm	6224 (31.8%)	5905 (31.2%)	319 (46.1%)	
Hand/Wrist	582 (3.0%)	574 (3.0%)	8 (1.2%)	
Leg	5 (0.0%)	5 (0.0%)	0 (0.0%)	
Other	1051 (5.4%)	1034 (5.5%)	17 (2.5%)	
Not documented	0	0	0	
**PIVC Outcomes**				
Prop Dwell to Stay				0.037^1^
Mean	0.68 (0.35)	0.680 (0.35)	0.71 (0.34)	
IV attempts				<0.001^1^
Mean	1.36 (0.09)	1.37 (0.00)	1.15 (0.42)	
IV wait time				0.071^1^
Mean	1.98 (2.71)	1.99 (2.74)	1.80 (1.68)	
Complications				0.093^2^
Infiltration/Leaking	3323 (87.0%)	3204 (86.9%)	119 (87.5%)	
Occlusion	499 (13.0%)	482 (13.1%)	17 (12.5%)	
Not documented	15777	15221	556	
**PIVC Cost ($)**				
IV attempts				<0.001^1^
Mean	47.68 (3.08)	47.95 (0.00)	40.29 (14.58)	
IV wait time low				0.071^1^
Mean	41.23 (56.50)	41.37 (57.13)	37.42 (35.05)	
IV wait time high				0.071^1^
Mean	237.77 (325.85)	238.58 (329.48)	215.78 (202.14)	
Complications				0.893^1^
Mean	11.68 (24.09)	11.68(24.08)	11.80 (24.22)	
OSTICK training cost				<0.001^1^
Mean	0.04 (0.22)	0.00 (0.00)	1.17 (0.00)	
Total cost low				<0.001^1^
Mean	100.63 (61.72)	101.00 (62.20)	90.68 (45.76)	
Total cost high				0.021^1^
Mean	297.17 (327.00)	298.20 (330.57)	269.04 (204.84)	

Abbreviations: BMI = body mass index; PIVC = peripheral intravenous catheter

*For continuous variables, mean (standard deviation) were presented. For categorical variables, frequencies (percentage) were presented.

^1^T-test

^2^Chi-square test

### Cost-effectiveness and incremental net benefit analyses

**Fig 1:**
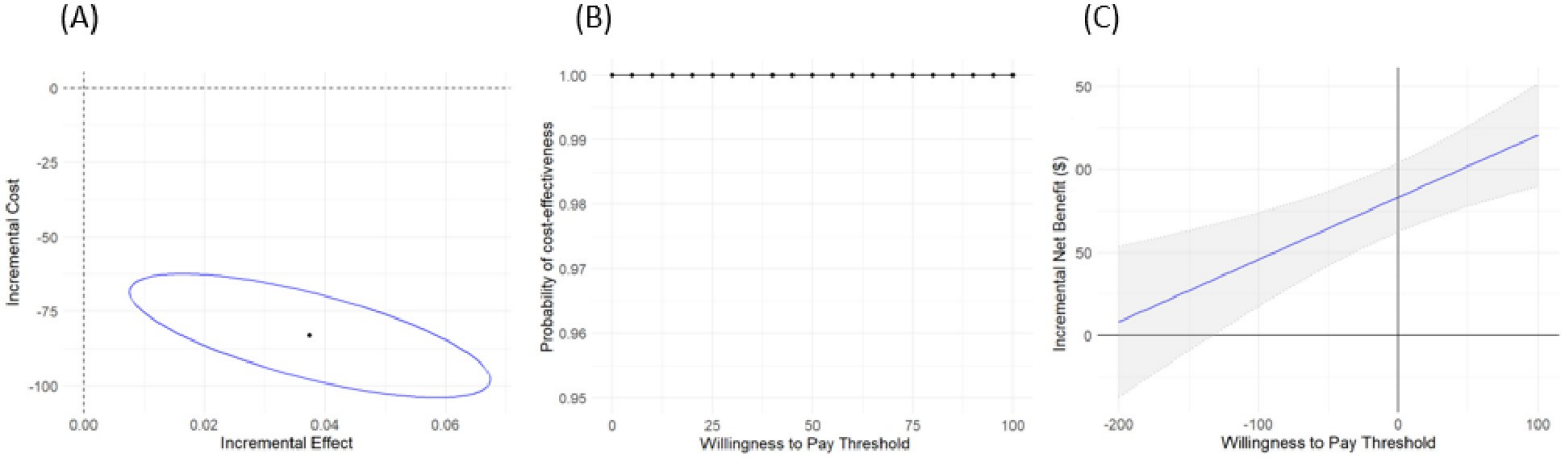
Cost-effectiveness of the OSTICK group compared with the non-OSTICK group: (A) the incremental costs (y-axis) and the incremental effects (x-axis) estimates on the cost-effectiveness plane, along with the 95% confidence ellipse, depicting that the adjusted estimates were in economically attractive areas. (B) the cost-effectiveness acceptability curve, showing the probability of the OSTICK being cost-effective across willingness-to-pay thresholds. (C) estimates of the incremental net benefit assessed as a function of willingness to pay, along with the 95% confidence intervals.

Table 4 presents regression estimates for multiple linear regressions of cost and effect. The estimate of incremental cost (ΔC) for each patient was -$83.175 (95% CI: -$103.953 to -$62.398; p<0.001), indicating that the OSTICK group saves money compared to the non-OSTICK group. The OSTICK group is also more effective at increasing the proportion of catheter dwell time relative to hospital length of stay (ΔE), with an estimate of 0.037 (95% CI: 0.016 to 0.059; p<0.001), compared to those in the non-OSTICK group. The estimated incremental cost-effectiveness ratio (ICER) for the OSTICK group compared with the non-OSTICK group was −$221.964 (95% CI: -$177.400 to -$381.716) per ten percentage points of PIVC dwell time to hospital length of stay increase. To illustrate the influence of these findings on the numerator and denominator of the ICER and their overall impact on cost-effectiveness, Fig 1A illustrates 95% CIs for the estimation of ΔC and ΔE. The south-east direction indicates the Operation STICK training appears more cost saving and more effective.

**Table 4:** Regression estimates of ΔC, ΔE, and ICER.

Terms	Estimate* (95% CI)	P Value
ΔC ($US)	-83.175 (-103.953, -62.398)	<0.001
ΔE	0.037 (0.016, 0.059)	<0.001
ICER	-221.964 (-177.400, -381.716)	

Abbreviations: CI = confidence intervals, ΔC = incremental cost, ΔE = incremental effect, ICER = incremental cost-effectiveness ratio

* ΔC and ΔE were estimated from multivariable linear regressions, adjusted for age, race, gender, BMI, Charlson Comorbidity Index, ESI, and insertion method. ICER was calculated as $\frac{\Delta C}{\Delta E}\times 0.1$

The cost effectiveness acceptability curve (CEAC) shown in Fig 1B indicates that if the decision-maker was willing to pay (WTP) $100 per ten percentage points increase of PIVC dwell time to hospital length of stay, there was a 100% chance that OSTICK training group is cost-effective compared with non-OSTICK group. Fig 1C illustrates that as WTP increases along the horizontal axis, the positive slope indicates OSTICK was more effective; at WTP = $0, the incremental net benefit (INB) $83 was positive, indicating OSTICK was less costly. The INB estimate was always positive for WTP > $0, suggesting OSTICK was estimated to be cost-effective, regardless of the decision-maker’s willingness to pay.

### Sensitivity analysis: Variation in IV wait time cost

To assess the robustness of our findings, we conducted a sensitivity analysis by varying the cost of IV wait time. In this scenario, we assumed the IV wait time cost to be $20.83 per hour, instead of the previously assumed $120.13 per hour. The results of this sensitivity analysis were consistent with our primary analysis.

S2 Table presents regression estimates for multiple linear regressions of cost and effect. The estimate of incremental cost (ΔC) was -$24.403 (95% CI: -$28.305 to -$20.502; p<0.001), indicating that the OSTICK group saves money compared to the non-OSTICK group. Similarly, the OSTICK group remained more effective at increasing the proportion of catheter dwell time relative to hospital length of stay (ΔE), with an estimate of 0.037 (95% CI: 0.016 to 0.059; p<0.001), compared to those in the non-OSTICK group. The ICER for the OSTICK group compared with the non-OSTICK group remained consistent at -$65.125 (95% CI: -$48.304 to -$125.422) per ten percentage points increase of PIVC dwell time to hospital length of stay.

The influence of these findings on the cost-effectiveness plane and the CEAC also remained similar to the primary analysis, as illustrated in Fig 1A and 1B, respectively. In Fig 1C, the INB estimate continued to be positive across all WTP thresholds, suggesting that the OSTICK intervention remained cost-effective, regardless of the decision-maker’s WTP. Overall, the sensitivity analysis corroborated the robustness of our findings regarding the cost-effectiveness of the OSTICK intervention.

### Subgroup analysis: DIVA and non-DIVA

Table 5 presents regression estimates for multiple linear regressions of cost and effect separating DIVA and non-DIVA cases. The estimate of incremental cost (ΔC) for each DIVA patient was -$164.291 (95% CI: -$218.815 to -$109.766; p<0.001), indicating that the OSTICK group saves money compared to the non-OSTICK group. The OSTICK group is also more effective at increasing the proportion of catheter dwell time relative to hospital length of stay (ΔE), with an estimate of 0.052 (95% CI: 0.015 to 0.088; p = 0.006), compared to those in the non-OSTICK group. The estimated incremental cost-effectiveness ratio (ICER) for the OSTICK group compared with the non-OSTICK group was −$317.038 (95% CI: -$247.438 to -$721.729) per ten percentage points of PIVC dwell time to hospital length of stay increase.

**Table 5:** Regression estimates of ΔC, ΔE, and ICER (subgroup analysis).

Terms	DIVA (US-Guided)		Non-DIVA (Traditional)	
	Estimate* (95% CI)	P Value	Estimate* (95% CI)	P Value
**Primary Results**				
ΔC ($US)	-164.291 (-218.815, -109.766)	<0.001	-37.849 (-62.338, -13.360)	0.003
ΔE	0.052 (0.015, 0.088)	0.006	0.028 (0.002, 0.055)	0.037
ICER	-317.038 (-247.438, -721.729)		-133.838 (-113.588, -795.688)	
**Sensitivity Analysis: Variation in IV Wait Time Cost** ^‡^				
ΔC ($US)	-46.010 (-55.950, -36.068)	<0.001	-11.847 (-16.474, -7.219)	<0.001
ΔE	0.052 (0.015, 0.088)	0.006	0.028 (0.002, 0.055)	0.037
ICER	-88.786 (-63.2698, -237.153)		-41.892 (-30.018, -429.971)	

Abbreviations: CI = confidence intervals, ΔC = incremental cost, ΔE = incremental effect, ICER = incremental cost-effectiveness ratio

* ΔC and ΔE were estimated from multivariable linear regressions, adjusted for age, race, gender, BMI, Charlson Comorbidity Index, and ESI. ICER was calculated as $\frac{\Delta C}{\Delta E}\times 0.1$

^‡^ IV wait time cost was assumed to be $20.83 per hour, instead of the primarily assumed $120.13 per hour.

The estimate of incremental cost (ΔC) for each non-DIVA patient was -$37.849 (95% CI: -$62.338 to -$13.360; p = 0.003), indicating that the OSTICK group saves money compared to the non-OSTICK group. The OSTICK group is also more effective at increasing the proportion of catheter dwell time relative to hospital length of stay (ΔE), with an estimate of 0.028 (95% CI: 0.002 to 0.055; p = 0.037), compared to those in the non-OSTICK group. The estimated incremental cost-effectiveness ratio (ICER) for the OSTICK group compared with the non-OSTICK group was −$133.838 (95% CI: -$113.838 to -$795.688) per ten percentage points of PIVC dwell time to hospital length of stay increase.

## Discussion

This study underscores the significant benefits of the Operation STICK vascular access program from both a cost and effectiveness perspective. Importantly, costs from inadequate vascular access begin accruing upon a patient’s entry into the ED and Operation STICK has demonstrated its ability to efficiently reduce both initial and subsequent costs and complications, extending its impact into inpatient care. As most patients in the ED require a PIVC for diagnostics and treatment, the cost savings of $83.18 per patient, extrapolated across thousands of cases, translates into significant financial benefits. For example, at the study site with 120,000 visits, cost savings amounted to $9.9M USD annually. Depending on the volume of a given department, these savings could range from hundreds of thousands to millions of dollars each year.

Historically, the costs related to inadequate PIVC care have been largely overlooked and ignored due to the complexities involved in managing PIVCs. While the initial placement of the PIVC may take place in the ED, subsequent management duties are frequently delegated to numerous personnel (shift changes, breaks, and diagnostic procedures) across various units and departments. This fragmentation makes it challenging to standardize, track, and audit PIVC care and implement solutions effectively. Each instance of PIVC access or assessment presents an opportunity to prevent complications or, conversely, increases the risk of complications. Despite published standards for PIVC management, real-world practice often lacks standardization, leading to fragmented and non-compliant care practices that elevate the risk of PIVC-related complications and associated costs [10]. Thus, it is not surprising that the majority of PIVCs fail due to complications [3]. Importantly, complications associated with PIVCs can impose significant financial burdens, with one cost modeling study indicating that for every 10,000 catheters utilized, complications of the index PIVC contribute to an excessive expense of $475,000 [7]. Given that the majority of hospital admissions originate in the ED, the Operation STICK program wisely targets the point of insertion of the initial PIVC. Through comprehensive education and training, Operation STICK has streamlined the insertion process, resulting in the placement of resilient PIVCs. These PIVCs demonstrate remarkable durability, enduring the challenges of inpatient care with remarkable longevity and minimal complications for the majority of hospitalizations. Ultimately, Operation STICK has fostered a cultural change, prompting ED clinicians to prioritize PIVC care for the entire hospitalization period, leading to improved functionality downstream and decreased costs due to fewer complications.

The significance of efficiently securing early IV access in the ED encounter cannot be overstated, considering that most ED patients require a PIVC for diagnostic blood studies, therapies, and radiological testing initiation [26]. At a juncture where ED efficiency is under scrutiny due to issues of overcrowding, ballooning wait times, and staffing constraints, a comprehensive approach is necessary to alleviate barriers that increase ED length of stay [26]. Even marginal reductions in time per encounter can lead to substantial enhancements in operational metrics, decreasing throughput times, left without being seen (LWBS) rates, and ambulance diverted traffic. Research demonstrates that a mere one-hour reduction in ED boarding time would result in additional daily revenue of $13,298 or annual revenue of $4.85 million from capturing LWBS and ambulance diverted traffic [27]. Specific to the operational impact of vascular access, a recent publication found that DIVA patients had a significantly greater time to obtain access (≥ 60 minutes), longer wait times for CT, and increased disposition times (≥ 240 minutes) all negatively impacting financial metrics [28]. In addition to more efficient PIVC placements, this study illustrates that more effective education led to fewer insertion attempts enhancing patient satisfaction, reducing wasted supplies, and allowing nurses more time to focus on other patient care tasks [28]. Overall, OSTICK reduced the time to PIVC placement, a core foundational component of care, by over 60 minutes, with an average of just 1.15 and 1.27 insertion attempts for non-DIVA and DIVA populations, respectively.

While this investigation captured costs across the continuum of the hospitalization, it is important to recognize that this analysis likely underestimates the cost savings achieved with OSTICK. While common complications are included in the cost equation, the modeling does not include the cost related to treatment delays due to these complications. In one randomized controlled trial, it was reported that on average it took 86 minutes to remove and replace an unexpectedly failed PIVC [29]. This time interval represents the delay due to a straightforward traditionally placed PIVC. When failure occurs in DIVA cases, the cost calculation can change dramatically as replacement of the failed PIVC can take many hours, often requires consultation of specialty vascular access services, and even upgrade to more advanced, invasive high-risk devices such as midline catheters or central venous catheters [30]. This study also tracks data limited to the initial PIVC complication. It is likely that some cases had multiple complications and failures and the cost implications of these complications was not included in this analysis. Literature shows that the likelihood of failure increases with each subsequent catheter insertion [31]. As complications affect the less reliable non-OSTICK catheters to a greater degree than OSTICK PIVCs, it follows that costs related to treatment delays, subsequent failures, and need for advanced rescue devices likely further inflate expenses in the non-OSTICK cohort.

## Limitations

This study is not without limitations. The retrospective nature of this study is a barrier as the study design cannot determine causality. Further, the use of the EHR as the data source has its constraints. While there is heavy reliance on accuracy and compliance with documentation with this study method, the literature suggests documentation of vascular access variables can be poor [32]. Nevertheless, this method allows for analysis of a large volume of cases. In particular, the accuracy of the time stamp provided in the medical record may not be reflective of the exact time the PIVC was placed. However, as each patient room is equipped with a desktop for patient care activities, nursing staff typically document PIVC details immediately after insertion. Further, this limitation likely impacts both groups equally. Finally, it is important to acknowledge that the findings of this study may not be generalizable to other settings given variations in healthcare practices, documentation standards, patient demographics, and acuity.

## Conclusions

Education and training in vascular access is an important component of delivering the highest quality of patient care. While formalized programming (Operation STICK) has tangible costs, the strategic investment in vascular access education and training can yield impressive financial returns. It is imperative for organizations to recognize the significant impact of such initiatives and prioritize the implementation of comprehensive programs.

## Supporting information

S1 Table Regressions of ΔC, ΔE, and INB. (DOCX)

S2 Table Regression estimates of ΔC, ΔE, and ICER (sensitivity analysis). (DOCX)
