# Supplementary material for: Cost effectiveness of a vascular access education and training program for hospitalized emergency department patients
Source: PLoS One. 2024 Oct 1;19(10):e0310676. doi: 10.1371/journal.pone.0310676 (PMC11444384; doi:10.1371/journal.pone.0310676)
Supplement: S1 Table — (DOCX) [file pone.0310676.s001.docx]

Supplementary Table 1. Regressions of ΔC, ΔE, and INB

| Dependent Variable | Regression Equation^*^ | Parameter | Estimate |
| --- | --- | --- | --- |
| Cost | $C=\alpha_{0}+\alpha_{1}OSTICK+\sum_{i=2}^{j} \alpha_{i}{Covariate}_{i}+\varepsilon$ | $\alpha_{1}$ | ΔC |
| Effect | $E=\beta_{0}+\beta_{1}OSTICK+\sum_{i=2}^{j} \beta_{i}{Covariate}_{i}+\varepsilon$ | $\beta_{1}$ | ΔE |
| Net benefit | $NB=\gamma_{0}+\gamma_{1}OSTICK+\sum_{i=2}^{j} \gamma_{i}{Covariate}_{i}+\varepsilon$ | $\gamma_{1}$ | INB |

Abbreviations: ΔC= incremental cost, ΔE= incremental effect, INB= incremental net benefit

*${Covariate}_{i}$ ​represents age, race, gender, BMI, Charlson Comorbidity Index, ESI, and insertion method, where $i$ ranges from 2 to $j$, and $j$ is the total number of covariates in the equation.
