# Supplementary material for: Cost effectiveness of a vascular access education and training program for hospitalized emergency department patients
Source: PLoS One. 2024 Oct 1;19(10):e0310676. doi: 10.1371/journal.pone.0310676 (PMC11444384; doi:10.1371/journal.pone.0310676)
Supplement: S2 Table — (DOCX) [file pone.0310676.s002.docx]

Supplementary Table 2. Regression Estimates of ΔC, ΔE, and ICER (Sensitivity Analysis)

| Terms | Estimate* (95% CI) |  | P Value |
| --- | --- | --- | --- |
| ΔC ($US) | -24.404 (-28.305, -20.502) |  | <0.001 |
| ΔE | 0.037 (0.016, 0.059) |  | <0.001 |
| ICER | -65.125 (-48.304, -125.422) |  |  |

Abbreviations: CI= confidence intervals, ΔC= incremental cost, ΔE= incremental effect, ICER= incremental cost-effectiveness ratio

* ΔC and ΔE were estimated from multivariable linear regressions, adjusted for age, race, gender, BMI, Charlson Comorbidity Index, ESI, and insertion method. ICER was calculated as $\frac{\Delta C}{\Delta E}\times0.1$
